# Supplementary material for: Protocol for assessing stakeholder engagement in the development and evaluation of the Informed Health Choices resources teaching secondary school students to think critically about health claims and choices
Source: PLoS One. 2020 Oct 12;15(10):e0239985. doi: 10.1371/journal.pone.0239985 (PMC7549807; doi:10.1371/journal.pone.0239985)
Supplement: Attachment — Submitted filename: Response Letter- 2020 03 30.docx [file pone.0239985.s001.docx]

Dear Editor,

Re: Response to your comments; for Manuscript ID PONE-D-20-04939, entitled “Protocol for evaluating stakeholder engagement in the design, evaluation, and dissemination of the Informed Health Choices resources for helping secondary school students learn to think critically about health claims and choices”.

Thank you very much for your timely response to our submission.

We have now carefully reviewed and addressed the comments as detailed below;

1. For studies reporting research involving human participants, PLOS ONE requires authors to confirm that this specific study was reviewed and approved by an institutional review board (ethics committee) before the study began. Please provide the specific name of the ethics committee/IRB that approved your study, or explain why you did not seek approval in this case.
   Once you have amended this/these statement(s) in the Methods section of the manuscript, please add the same text to the “Ethics Statement” field of the submission form (via “Edit Submission”).

Response: Ethics approval has been sought from the respective national ethics approval committees in all the three countries. This has been addressed in more detail on page 13.

2. Please provide additional details regarding the methods used in this study. For instance, it’s not clear what your sites are and how they will be selected and the interview or survey guidelines and materials. Understanding the content, development and validation of this questionnaire is important before we proceed. As per the Registered Reports framework, we require that the protocol should be detailed enough that by reading it we should already have an idea about whether the final paper should be accepted or not.

Response: Additional details regarding the methods used in this study have been availed on pages 08, 09, 10 and 11.
